# Ureolytic/Non-Ureolytic Bacteria Co-Cultured Self-Healing Agent for Cementitious Materials Crack Repair

**DOI:** 10.3390/ma11050782

**Published:** 2018-05-11

**Authors:** Hyeong Min Son, Ha Yeon Kim, Sol Moi Park, Haeng Ki Lee

**Affiliations:** Department of Civil and Environmental Engineering, Korea Advanced Institute of Science and Technology, 291 Daehak-ro, Yuseong-gu, Daejeon 34141, Korea; nemilhm@kaist.ac.kr (H.M.S.); gkdus305@kaist.ac.kr (H.Y.K.); solmoi.park@kaist.ac.kr (S.M.P.)

**Keywords:** ureolytic bacteria, non-ureolytic bacteria, co-cultured bacteria, CaCO_3_ precipitation, self-healing agent

## Abstract

The present study investigated the CaCO_3_ precipitation performance of ureolytic and non-ureolytic bacteria co-cultured as a self-healing agent for cementitious materials crack repair. Three different inoculum ratios of ureolytic *Sporosarcina pasteurii* and non-ureolytic *Bacillus thuringiensis* (10:0, 8:2, or 5:5) were used. The effect of coculturing ureolytic and non-ureolytic bacteria on microbial metabolism was investigated by measuring the rate of growth in urea-containing medium and the rate of NH_4_^+^ and CaCO_3_ production in urea–calcium lactate medium. The self-healing efficiency of co-cultured bacteria was examined by exposing cement mortar specimens with predefined cracks to media containing single urease-producing or co-cultured bacteria. The obtained results provide new findings, where CaCO_3_ precipitation is improved by co-culturing ureolytic and non-ureolytic bacteria, owing to the relatively faster growth rate of non-ureolytic bacteria. The crack filling rate correlated with the width of crack, in particular, specimens with a smaller crack width showed the faster filling effect, indicating that the crack width can be a dominant factor influencing the CaCO_3_ precipitation capacity of co-cultured bacteria.

## 1. Introduction

Concrete microcracks are a natural phenomenon and occur owing to many factors, such as changes in external force and drying shrinkage [1,2]. The formation of microcracks weakens the mechanical properties of concrete and makes the transport of moisture and chemicals through the matrix easier, accelerating concrete deterioration [3,4,5,6,7]. The durability performance of reinforced concrete structures is highly dictated by the ability to resist against such material transport. For example, sulfate ions penetrate through microcracks and cause internal pore expansion due to ettringite formation, known as sulfate attack [8,9], and chloride ions oxidize steel to accelerate the corrosion of steel bars embedded in concrete [10,11,12]. Repair work is mandatory to mitigate this problem and to extend the service life of concrete structures. While this often involves chemical repair materials (i.e., epoxy resins, polyesters, and polyurethane) [13,14], fundamental problems remain owing to difficulties in precisely locating microcracks, use of chemicals for healing microcracks being environmentally harmful, and the repair effect not being continuously maintained [15,16].

Against this backdrop, self-healing concrete has received significant attention over recent years owing to the potential of repair/maintenance-free concrete with the ability to heal microcracks, deemed as the main source of deterioration in concrete structures [17,18,19,20,21,22,23]. Bacteria incorporated in self-healing concrete act as healing agents, healing by precipitating CaCO_3_ at the cracked zone [3,4,17,18,19,22,24,25,26,27]. Self-healing concrete has a mechanism to heal cracks through forming CaCO_3_ by binding to CO_3_^2−^ formed by urea decomposition of bacteria and Ca^2+^ in concrete [28]. As a result, urea decomposition performance of bacteria is essential. 

Accordingly, studies on self-healing concrete using bacteria preceded the employment of ureolytic bacteria as a self-healing organism [23]. For instance, Bang et al. reported that CaCO_3_ precipitated by decomposition of urea by ureolytic bacteria was effective to improve the strength and to repair microcracks of concrete [29]. A similar result was obtained by Ghosh et al., who reported an increase in the strength of mortar specimens when *Shewanella* species were used [30]. The increased strength of concrete induced by CaCO_3_ precipitation of *Bacillus sphaericus*, another known type of ureolytic bacteria, was also found to be accompanied by an enhancement in durability performance, compared with conventional treatment methods [31]. Self-healing induced by bacteria is not only limited to conventional concrete (i.e., those using Portland cement as a binder), but is also known to occur in fly ash-based concrete. This was intensively investigated by Chahal et al. [32], which found that compressive strength, water absorption and chloride permeability were affected by the added amount of *S. pasteurii*. Another aspect of self-healing concrete being widely studied concerns enhancing the survival rate of bacteria in the cement matrix, since it is directly linked to the self-healing capacity. One way to achieve this is to incorporate bacterial spores directly into concrete during the mixing process, along with a calcium source and urea [19]. In addition, microcapsulation of bacterial spores was found to be effective for ensuring the survival of the bacteria despite the internal pressure caused by concrete hardening when the bacteria spore is incorporated [19,22]. 

Despite a considerable number of studies investigating various means to improve the self-healing performance of bacteria, these studies used a single urease-producing bacterium, implying that there remains plenty of room for improvement with regards to microbial metabolism. In addition, in a self-healing concrete with bacteria, the limitations of healing effects with respect to crack width and crack generation time have been studied. In a self-healing concrete with bacteria, the limitations of healing effects with respect to crack width and crack generation time have been studied. Luo et al. reported that it is difficult to heal cracks of ≥0.8 mm and that it is hard to heal the crack as the crack width increases [25]. In addition, they showed that the crack healing effect is highly reduced when crack generation time is more than 60 days [25]. Considering these limitations on the crack healing performance with ureolytic bacteria, further studies are necessary to improve the crack healing rate. To this end, the present study investigated the self-healing agent using co-cultured bacteria as an effective means to improve microbial metabolism, thereby enhancing the CaCO_3_ precipitation efficiency.

## 2. Experimental Procedure

### 2.1. Bacterial Strains and Growth Medium

*Sporosarcina pasteurii* (ATCC 11859) and *Bacillus thuringiensis* (ATCC 10792) were the bacteria used in this study. *S. pasteurii*, a urease-producing organism, is known to survive in high alkaline environments of concrete [33], and decomposes urea (CH_4_N_2_O) into NH_4_^+^ and HCO_3_^−^ to precipitate CaCO_3_ in the presence of Ca^2+^ [26]. *B. thuringiensis*, a non-urease producing organism, is a gram-positive bacteria that has a negative charge on the outer cell wall and can absorb Ca^2+^ [34]. However, this bacterial species cannot precipitate CaCO_3_ on its own because it does not decompose urea. For the preculturing of bacteria, *S. pasteurii* (obtained from the Korean Collection for Type Cultures, Jeongeup, South Korea) was added to 30 g/L Tryptic Soy Broth (Soybean–Casein Digest Medium) (TSB, Becton Dickinson, Franklin Lakes, NJ, USA) supplemented with 2% urea (333 mM) and cultured at 30 °C by shaking at 200 rpm in 650 mL cell culture flasks (SPL Life Science, Pocheon, South Korea) sealed with filter caps for sterile gas exchange. TSB was autoclaved at 121 °C and 1.5 psi for 20 min for sterilization, and the urea was filtered with a 0.22 μm pore size filter to prevent urea decomposition at high temperatures. *B. thuringiensis* (obtained from the Korea Collection for Type Cultures, Jeongeup, South Korea) was shake-cultured in 650 mL cell culture flasks (SPL Life Science, Pocheon, South Korea) sealed with filter caps for sterile gas exchange at 30 °C and 200 rpm in sterilized 25 g/L Miller’s Luria–Bertani broth (LB broth, Becton Dickinson, Franklin Lakes, NJ, USA).

### 2.2. Experimental Details

Three different inoculum ratios were employed to investigate the effect of co-culture. *S. pasteurii* alone was used as a control, and *S. pasteurii* and *B. thuringiensis* were inoculated at a ratio of 8:2 and 5:5, respectively. These ratios are hereinafter referred to as A, B, and C, respectively, for simplicity (Table 1). *S. pasteurii* and *B. thuringiensis*, having reached the exponential phase in the preculture, were inoculated at 0.5% in medium at A, B, and C ratios. Optical Density (OD) was measured using the Genesystm 30 Visible Spectrophotometer (Thermo Fisher Scientific, Waltham, MA, USA) at a wavelength of 600 nm to determine the growth pattern of the two species according to the inoculation ratios of A, B, and C. To observe the growth curve of bacteria at the tested inoculum ratios, the medium comprised 30 g/L TSB supplemented with 2% urea (333 mM) and was cultured in 650 mL cell culture flasks (SPL Life Science, Pocheon, South Korea) sealed with filter caps for sterile gas exchange at 30 °C with shaking at 200 rpm. Extraction of 1 mL of aqueous phase was performed every 4 h during 100 h across A, B, and C ratios. *S. pasteurii* and *B. thuringiensis* were incubated with Urea–TSB solid medium containing 3% TSB, 2% Urea, Urea agar base medium consisting of 1.8% Urea agar base and 1.8% agar for 1 day to confirm the antagonistic activity of the two bacteria. The crossing points of *S. pasteurii* and *B. thuringiensis* drawn in “M” and “=” shapes on the same medium were analyzed to investigate the effect of each bacteria on the growth of other bacteria.

The urea–calcium lactate medium was prepared for production of CaCO_3_. This medium contained 6 g/L Nutrient Broth (NB, Becton Dickinson, Franklin Lakes, NJ, USA) for the inclusion of various basic nutrient minerals, 333 mM urea (2%) for HCO_3_^−^ production, and 91.7 mM calcium lactate for the supply of Ca^2+^. NB was autoclaved, and the urea and calcium lactate were sterilized using a 0.22 μm pore size filter. The inoculum of *S. pasteurii* and *B. thuringiensis* was added to urea–calcium lactate medium in 650 mL cell culture flasks (SPL Life Science, Pocheon, South Korea) sealed with filter caps for sterile gas exchange at ratios of A, B, and C and was incubated at 30 °C by shaking at 200 rpm. 

To compare the ureolytic activity and the degree of Ca^2+^ consumption in CaCO_3_ production, the concentration of NH_4_^+^ produced by the urease expressed by *S. pasteurii* and the concentration of Ca^2+^ used in the production of calcium carbonate were measured. The concentration of NH_4_^+^ and Ca^2+^ dissolved in the aqueous phase in the culture solutions was measured using Metrohm 930 Compact IC Flex (Metrohm, Riverview, Herisau, Switzeland) and a cation exchange column. Five milliliters of the aqueous phase were extracted at an interval of 11 h for each of the tested ratios. Sampling was conducted until no further change in the NH_4_^+^ and Ca^2+^ concentration was observed. The supernatants of the extracted samples were obtained using a High Speed Refrigerated Centrifuge SUPRA 22K (Hanil Science Medical, Daejeon, South Korea) at 4800× *g*, 4 °C and then the NH_4_^+^ and Ca^2+^ concentration was analyzed. 

Changes in the pH of the culture medium due to NH_4_^+^ production from urea hydrolysis in *S. pasteurii* were measured using a SevenGo Portable pH meter (Mettler Tolredo, Columbus, OH, USA). Four milliliters of aqueous phase were extracted at an interval of 4 h for A, B, and C ratios in urea–calcium lactate medium. The initial pH of each culture was kept identical at 7.5.

The charge of microbial cell surface upon precipitation of calcium carbonate was investigated by zeta potential measurement. The bacterial species were incubated in urea–lactate medium at a co-culture ratio of A, B, and C, and 5 mL of aqueous phase was extracted after 11 h, 22 h, and 33 h. Each sample was diluted 10-fold and a well dispersed sample was measured on a Nano ZSP/ZEN5602 (Malvern Instrument, Malvern, UK).

The amount of CaCO_3_ produced with the different *S. pasteurii*: *B. thuringiensis* ratios was measured to compare the difference in production for different ratios. CaCO_3_ was recovered and quantified after shake-incubating in urea–calcium lactate medium at 30 °C and 200 rpm for 33 h. For CaCO_3_ quantitation, the culture broth was collected on Whatman filter paper No. 2. The remaining cells and culture medium were removed by washing the filter paper with distilled water, followed by drying at 105 °C for 2 h before measuring the weight. 

To compare the self-filling capacity of ratios A, B, and C, mortar specimens were prepared. Mortar specimens were fabricated with a water-to-cement ratio of 0.4 using a 5 × 5 × 5 cm^3^ mold. The mortar specimens were sealed with a plastic wrap after casting and were cured at room temperature for the initial 24 h to prevent water evaporation during setting, then cracks were introduced in the mortar specimens. Both the mortar specimens with predefined cracks and the culture container were sterilized by spraying with 70% ethanol. The sterile urea–calcium lactate medium for producing CaCO_3_ was placed in a culture vessel together with the mortar specimen, and the two species that reached the exponential phase were exposed to the culture vessel at ratios A, B, and C. The self-filling of cracks was observed at 500 magnification on a Digibird USB microscope ORT-500 (Digibird, Goyang, Korea) after 1 and 3 days of incubation at 30 °C. For comparing the effect of crack filling by co-culture with that of control, a bacteria-free urea–calcium lactate medium was prepared to cure mortar specimens as control. The other experimental procedures are the same except for the medium conditions.

The morphology of the co-cultured species and the precipitate formed around the bacteria were observed using a scanning electron microscope JSM-6300 (SEM, JEOL LTD, Akishima, Tokyo, Japan). Three samples with different co-culture ratios of the two species were shake-incubated in urea–calcium lactate medium for 33 h at 30 °C and 200 rpm. The precipitated CaCO_3_ was filtered through Whatman filter paper No. 2, collected, and dried at 105 °C in an oven to prepare powder samples for SEM (JEOL LTD, Akishima, Tokyo, Japan) and XRD (Rigaku, Tokyo, Japan) analyses. The XRD analysis was conducted using Rigaku D/MAX-2500 (Rigaku, Tokyo, Japan) with Cu-Kα radiation at a scan range of 5°–65° 2θ and a scan speed of 0.5°/min.

## 3. Results

### 3.1. Culture Growth

**Figure 1:**
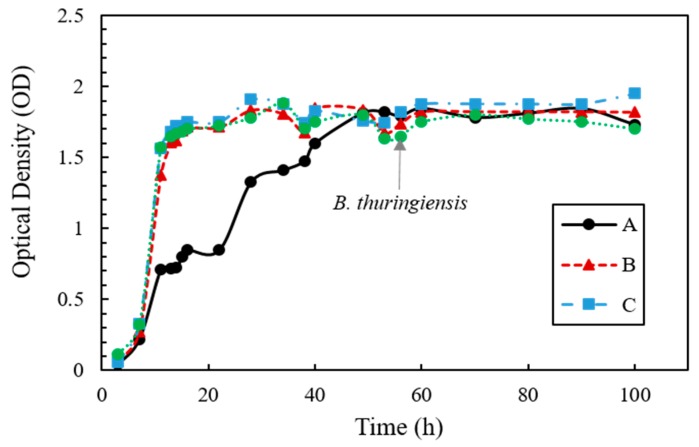
Optical density measurement for cell growth in urea–TSB medium over time.

The growth rate of the bacteria in urea–TSB medium with inoculum ratios of A, B, and C are shown in Figure 1. The growth for A, where *S. pasteurii* was cultured alone, showed an exponential phase between 7 and 40 h of incubation. For B and C, where *S. pasteurii* and *B. thuringiensis* were co-cultured at 8:2 and 5:5, respectively, the OD was 1.37 and 1.56 at 11 h, respectively, corresponding to a value two-fold higher than for A (0.71). The OD of C was slightly higher than B at almost all sampling points, and B and C reached their peak OD (1.83 and 1.90 respectively) at 30 h. *B. thuringiensis* showed rapid growth until the first 10 h, followed by *S. pasteurii*, and the OD increased at 30 h in B and C. The bacterial growth for all of the tested co-culture ratios (A, B, and C) reached the stationary phase after 40 h, showing similar OD values from this point onward. 

**Figure 2:**
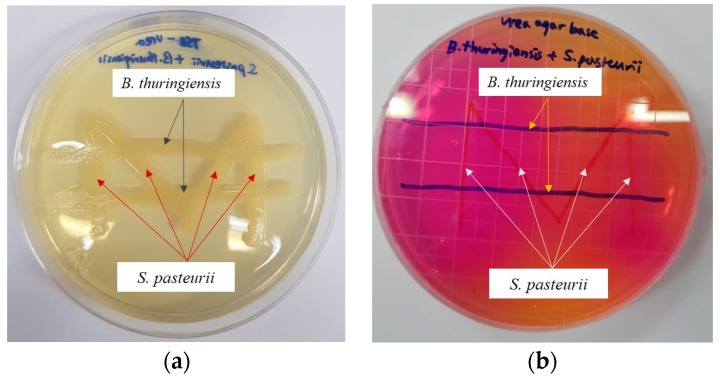
Bacteria growth images in: (**a**) urea–TSB solid medium; and (**b**) urea agar base medium.

Urea–TSB and Urea Agar base culture flasks cultured with *B. thurigiensis* and *S. pasteurii* are shown in Figure 2. Inhibition zones which occurs when antagonistic activity [35] is progressed were not found in both TSB-Urea and Urea agar base medium. It can be inferred that *B. thuringiensis* and *S. pasteurii* did not inhibit growth with each other. In addition, district red colors were observed by urea decomposition in Urea agar base medium. Since these red colors were observed in the co-cultured contacts of both bacteria (Figure 2), it can be said that non-ureolytic bacteria, *B. thuringiensis*, did not inhibit the ureolytic activity of *S. pasteurii*.

### 3.2. Dissolved NH_4_^+^ and Ca^2+^ Concentration

**Figure 3:**
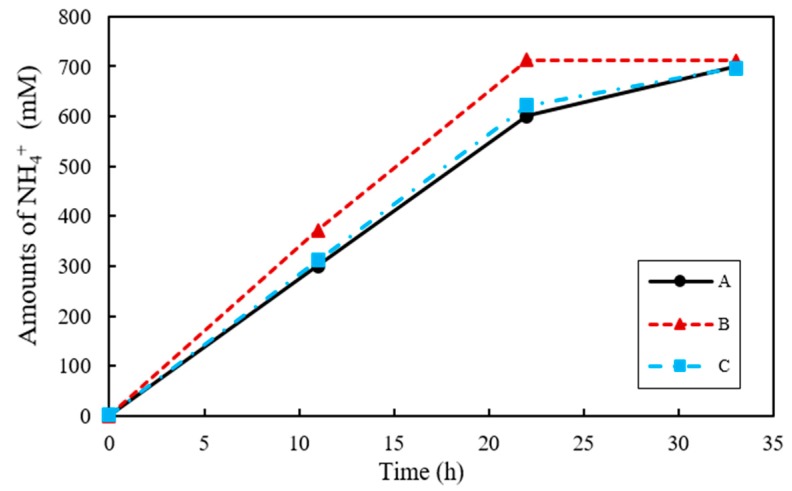
The changes of dissolved NH_4_^+^ concentration in urea–calcium lactate medium over time.

Urease is a common enzyme expressed by microorganisms, where 1 mol of urea (NH_4_CO_3_) is hydrated by urease to generate 2 mol of NH_4_^+^, 1 mol of HCO_3_^−^, and 2 mol of OH^−^ [36]. As a result, the pH of culture media increases, with HCO_3_^−^ converted to CO_3_^2−^ that then binds Ca^2+^ in the surroundings, and as a result CaCO_3_ is produced [37]. The urea degradation performance of the ureolytic bacteria *S. pasteurii* can be determined by measuring the concentration of NH_4_^+^ produced in the culture medium. The change in NH_4_^+^ concentration in each sample is shown in Figure 3. The NH_4_^+^ concentration for co-culture ratios A, B, and C reached 698.2, 711.1 and 696.3 mM at 33 h, respectively. However, the rate at which NH_4_^+^ was produced at 11 h was the highest for B at 371.4 mM, while those for A and C were 300.0 mM and 311.2 mM, respectively. Given that 1 mol of urea is decomposed into 2 mol of NH_4_^+^, 666 mM NH_4_^+^ was expected to be produced from 333 mM urea. Nevertheless, the amount of NH_4_^+^ produced was in fact greater than predicted at 104.83%, 106.77%, and 104.59% for A, B, and C, respectively, in relation to the amount of nitrogen initially supplied by the urea.

**Figure 4:**
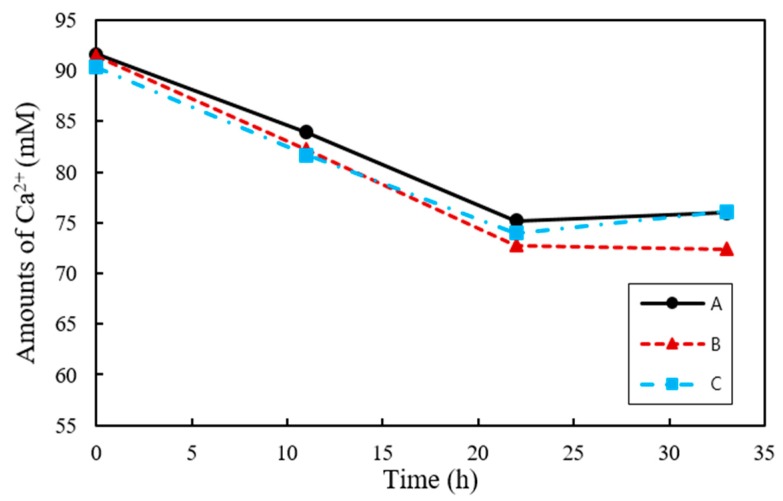
Ca^2+^ consumption by *S. pasteurii* and *B. thuringiensis* in urea–calcium lactate medium over time.

As urea is degraded by urease, CO_3_^2−^ is combined with Ca^2+^ in the culture solution to form CaCO_3_. Therefore, it is possible to deduce the amount of Ca^2+^ used in the production of CaCO_3_ by measuring the concentration of the remaining Ca^2+^. At 33 h the Ca^2+^ consumption was highest for B at 19.12 mM, followed by A and C at 16.44 and 14.25 mM, respectively (Figure 4). The Ca^2+^ concentrations showed no further change after 22 h and were not entirely exhausted in all samples.

### 3.3. CaCO_3_ Precipitation, Ph Change and Zeta Potential

**Figure 5:**
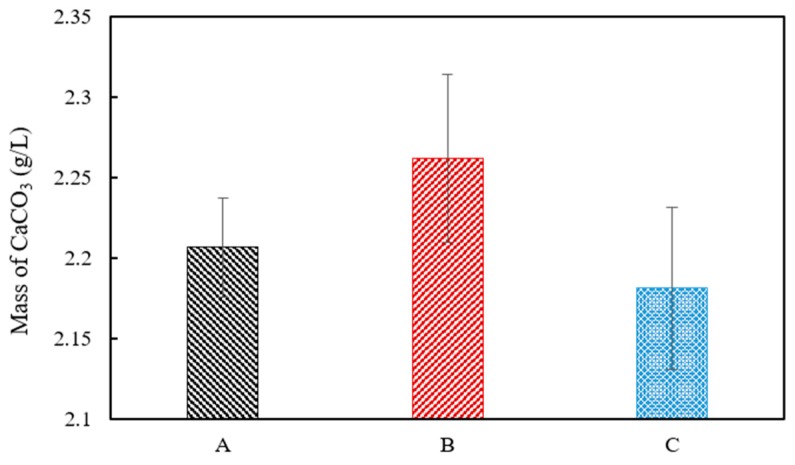
Dry weight of precipitated CaCO_3_ at urea–calcium lactate medium at 33 h.

**Figure 6:**
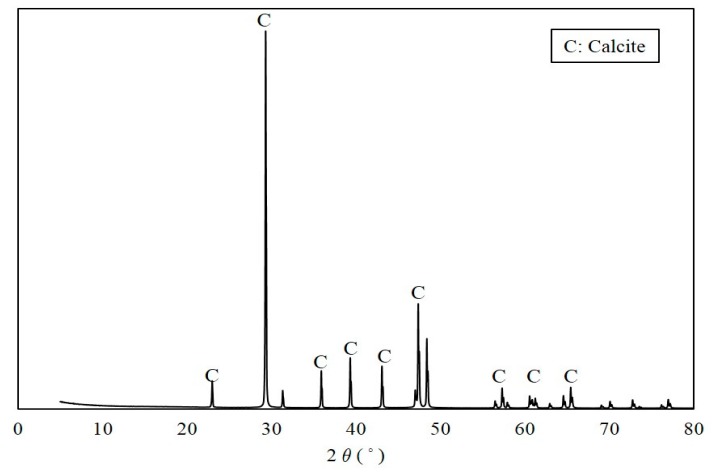
XRD pattern of CaCO_3_ precipitated in urea–calcium lactate medium at 33 h.

A white precipitate that formed in the urea–calcium lactate medium after culturing for 33 h was retrieved, and its weight was measured (Figure 5). The data values and error bars represent the averages and standard derivations of triplicate experiments. The weight of CaCO_3_ precipitated was the highest from ratio B cultures with *S. pasteurii* and *B. thuringiensis* at ratio of 8:2 (2.26 ± 0.05). The weight of recovered CaCO_3_ from ratio A and C cultures was 2.20 ± 0.03 and 2.18 ± 0.05, respectively. The weight of CaCO_3_ precipitated from ratio B cultures with a *S. pasteurii*:*B. thuringiensis* ratio of 8:2 was found to have the greatest weight. This is in close agreement with the Ca^2+^ consumption provided in Figure 4 at 33 h, showing that Ca^2+^ consumption was highest for B at 19.03 mM, followed by A and C at 15.65 and 14.25 mM, respectively. The XRD pattern of the white precipitate shown in Figure 6 confirms the presence of calcite (CaCO_3_, PDF# 01-072-1937), with noticeable peak intensity at 28°–30° 2θ. 

**Figure 7:**
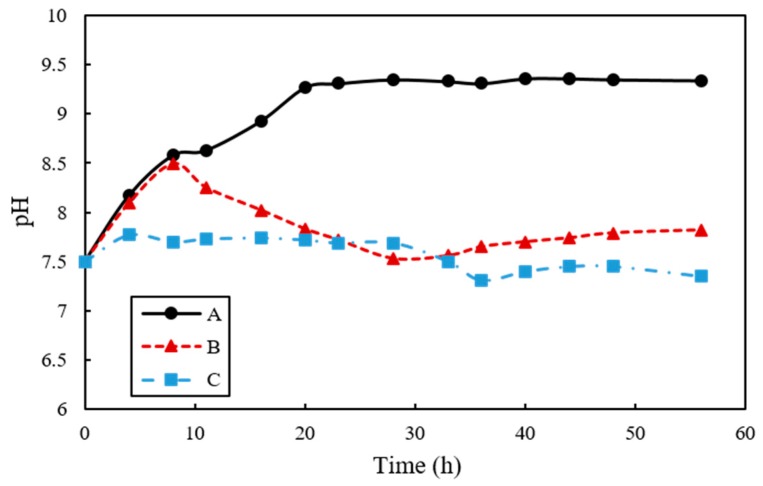
The changes of pH in urea–calcium lactate medium over time.

**Figure 8:**
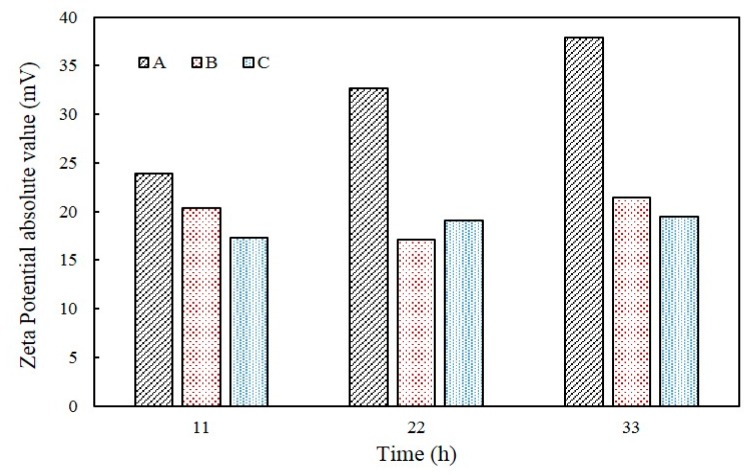
The changes of zeta potential in urea–calcium lactate medium over time.

The evolution of pH in A, B, and C samples are shown in Figure 7. The pH in A tended to increase from 7.5 to 9.31 until 24 h. On the other hand, for B samples the pH increased to 8.49 until 8 h and then decreased to 7.56 after 35 h. Lastly, C showed almost no change in pH and was maintained at approximately 7.6. The zeta potential absolute value of A increased, but B and C tended to be maintained. The value of pH of A increased from 8.63 to 9.31 and the zeta potentials ranged from −23.9 mV to −37.9 mV between 11 h and 33 h. The value of zeta potential of B is −20.4 mV, −17.1 mV, and −21.4 mV, respectively, in pH 8.25 (11 h), 7.72 pH (22 h), and 7.65 pH (33 h) and that of C showed continuous value around −19 mV from 11 h to 33 h (Figure 8).

**Figure 9:**
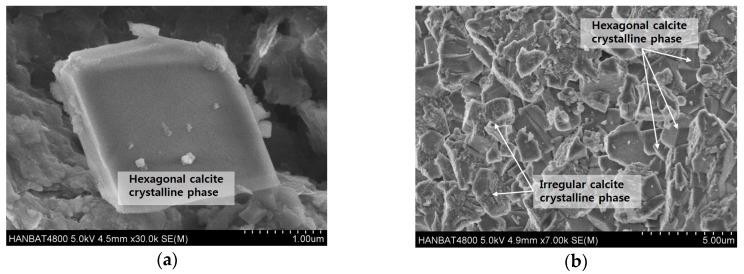
SEM images of calcite precipitated in the co-culture medium at magnification of: (**a**) 30,000; and (**b**) 7000.

SEM images of CaCO_3_ precipitated upon decomposition of urea by the bacteria are shown in Figure 9. A hexagonal crystalline phase and amorphous phase coexisted, and the crystal phase was tightly bonded (Figure 9a) and the hexagonal calcite crystals were clearly observable (Figure 9b). 

### 3.4. Effect of CaCO_3_ Precipitation in Crack on Cementitious Materials

**Figure 10:**
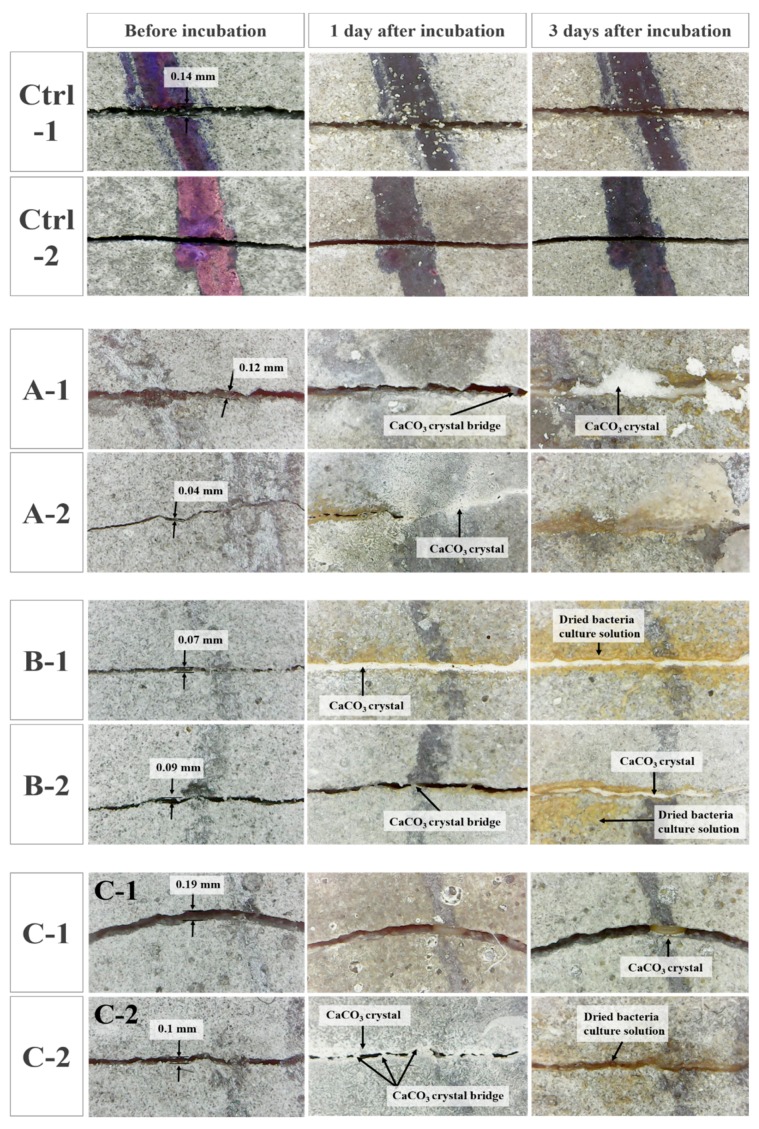
Microscopic observation of crack healing after one and three days of incubation.

The CaCO_3_ precipitation process in crack for the mortar specimens with predefined cracks in A, B, and C cultures is shown in Figure 10. It was observed that not only the composition ratio of the culture medium but also the shape of the cracks had a significant influence on the filling rate. For culture A-1 with a crack width of 0.12 mm, a CaCO_3_ precipitate was observed after three days of incubation. On the other hand, A-2 with a crack width of 0.04 mm showed the formation of CaCO_3_ and the crack filling even as early as one day of incubation. Similarly, B-1 showed complete crack filling performance after one day of incubation, and the dried bacteria and culture solution appeared prominently around the crack after three days of incubation. Meanwhile, the 0.19 mm wide crack combined with the C culture had the slowest rate of crack filling, showing scarce distribution of CaCO_3_ precipitates after three days of incubation. However, in the case of the C-2 mortar specimen with a 0.1 mm crack, filling of the crack had occurred due to deposition of CaCO_3_ after one day of incubation, with complete filling of the crack observed after three days of incubation. On the other hand, cracks in mortar specimens that was cured in bacteria-free urea–calcium lactate medium as control did not heal after one and three days.

## 4. Discussion

### 4.1. Microbial Metabolism of Co-Cultured Bacteria

As self-healing of concrete using bacteria relies on the formation of CaCO_3_ upon decomposition of urea by bacteria, it is essential to incorporate urea-decomposing bacteria. In addition, NH_4_^+^ and OH^−^ formed through urea decomposition of bacteria induced an increase in the pH by producing CO_2_ and created an environment favorable for precipitation of CaCO_3_ [38]. Therefore, most of the studies on self-healing concrete using bacteria use urea-decomposing bacteria. In contrast, little attention has been given to the self-healing potential of non-ureolytic bacteria and its application.

Obst et al. (2006) studied that the reduction of net negative electric potential of bacteria cell surface is due to the adsorption of Ca^2+^ in culture solution on the cell surface, suggesting that the cell surface of the bacteria provides a nucleation site for the CaCO_3_ precipitation [39]. In this study, the amount of Ca^2+^ dissolved in the culture medium was the smallest in B, while A and C showed a similar value. This result is consistent with B showing the highest amount of CaCO_3_ production, as indicated by weight measurements of the precipitated CaCO_3_. Both results show that cocultivation of *S. pasteurii* and *B. thuringiensis* has a synergetic effect on CaCO_3_ precipitation, attributed to the presence of more nucleation sites. High cell density during the hydrolysis of urea was effective in increasing the diversity and size of CaCO_3_ crystals. 

As *S. pasteurii* is a gram-positive bacteria, the surface of the cell wall is negatively charged by teichoic acid bound with phosphate, and Ca^2+^ in the culture solution is attracted to saturation around the cell wall [40]. Therefore, CaCO_3_ is produced as the Ca^2+^ present around bacteria reacts with CO_3_^2−^ formed upon urea degradation by the release of urease into the culture medium by *S. pasteurii*. However, as CaCO_3_ is formed around the cell membrane, the surface of *S. pasteurii* is saturated owing to continuous CaCO_3_ precipitation, effectively making it difficult for Ca^2+^ and urea to reach the surface of the bacteria. Consequently, the bacterial CaCO_3_ production activity is lowered. *B. thuringiensis* with a relatively faster growth rate may possess an enhanced binding capacity for Ca^2+^ and CO_3_^2−^ by providing additional nucleation sites outside of those provided by *S. pasteurii* embedded in CaCO_3_.

When *B. thuringiensis* and *S. pasteurii* are co-cultured, the concentrations of NH_4_^+^ and Ca^2+^ for CaCO_3_ precipitation were expected to vary according to urea degradation performance of bacteria. Since *S. pasteurii* was the only species capable of decomposing urea in this study, it was anticipated that the cultures that solely consist of *S. pasteurii* would display the highest concentration of NH_4_^+^ among the samples. However, the highest amount of NH_4_^+^ was observed in the B cultures at all sampling points during the measurement of NH_4_^+^ concentration. As shown in the OD results, *B. thuringiensis* had a relatively faster growth rate in comparison with *S. pasteurii*, more rapidly decomposing NB and thereby releasing additional NH_4_^+^. Consequently, the highest concentration of NH_4_^+^ was achieved in B cultures, where the amount of NH_4_^+^ was further increased by decomposition of NB as well as urea. 

*S. pasteurii* and *B. thuringiensis* were inoculated into the culture medium at a ratio of A, B, and C, but the ratio of the two species in a culture medium after incubation is different from the initial inoculation ratio because of the different growth rates of each bacterium. However, rapid and high production of CaCO_3_ is linked to the initial inoculation ratio, and it may be reasonable to assume that the change in the ratio of two bacteria after co-culturing does not affect the outcome of the rapid effect of crack healing. In Figure 1, the optical density value of sample A cultured with only *S. pasteurii* was twice as low as that of *B. thuringiensis*-cultured sample. However, although a difference was observed in NH_4_^+^ production between each sample inoculated at ratios of A, B, and C, there was no significant difference in NH_4_^+^ production in each sample with respect to the difference in OD value. In addition, despite of the slow growth rate, *S. pasteurii* provided sufficient CO_3_^2−^ by degrading urea, and the nucleation site increased owing to the rapid growth of *B. thuringiensis*. Therefore, ratio B culture produced higher amount of CaCO_3_ in a short time (Figure 3 and Figure 4). Hence, to increase the effect of crack healing, co-cultivation of *S. pasteurii* and *B. thuringiensis* at a ratio of 8:2 is more effective.

The evolution of pH was also observed to be different according to the samples used. For instance, the pH in A and B was increased to 8.5 within 10 h of incubation, probably due to the increased concentrations of NH_4_^+^ and OH^−^ that were produced by the hydrolysis of urea. However, the pH in B showed a sharp decrease after 10 h, presumably attributed to CaCO_3_ precipitation, where by water is released. Despite the decomposition of urea, the facultative anaerobic organism *B. thuringiensis* reduces culture medium pH due to the respiration process when oxygen is present, hence, the pH remained stationary and relatively unchanged in B and C that contained *B. thuringiensis* with a higher growth rate than *S. pasteurii*. It is interesting to note that the pH in C was constant from the beginning; even though the pH can be increased by increased OH^−^ concentration from the ureolytic process, this can be compensated by the breathing of CO_2_ and production of CaCO_3_ by *B. thuringiensis*. These results are also closely related to the zeta potential change of each sample. This implied that increasing degradation of urea had an effect on increasing pH, indicating that increasing the degree of dispersion result in increasing zeta potential absolute value.

### 4.2. Morphology of CaCO_3_ Precipitated by Co-Cultured Bacteria

SEM results showed various crystal phases that formed around the nucleation site of the bacteria. In addition, the crystal phases of calcite with a hexagonal shape and other phases with spherical shape were observed. It should be noted that the size and crystallinity of the crystal phase are affected by the concentration of urea and CaCO_3_ in the culture medium; as the concentration of urea and calcium increases, the crystal size and crystallinity becomes lower [40]. This led to the formation of calcite, as well as a variety of other phases that were amorphous in nature.

### 4.3. Crack-Filling Efficiency of Co-Cultured Bacteria

Numerous studies have been performed to determine the performance of self-healing concrete with respect to the healing time and crack width variable, and it has been shown that the healing rate reduces as crack width and healing time increase. In addition, because crack healing performance is influenced by the change in bacterial species and healing environment conditions, crack width and healing time should be investigated in crack healing by co-culturing, as reported in this study. The crack filling process of mortar specimens with predefined cracks via CaCO_3_ precipitation in culture media showed different rates of filling. The smaller the width of the crack, the faster the observed crack filling was. Similarly, crack filling was less apparent even after three days of exposure to the culture medium, when the width of the crack was sufficiently large. The results indicate that the crack width is an important factor in crack filling via CaCO_3_ precipitation. A similar result was found in a previous study that reported that the maximum crack filling capacity achieved in a mortar specimen exposed to medium containing 5% *B. sphaericus* spores was in the range of 330–400 nm [22]. 

In this study, CaCO_3_ precipitation and the effect of self-filling in the mortar by the coculturing of ureolytic and non-ureolytic bacteria were investigated. As a result, it might be assumed that the increase of the quantity of CaCO_3_ precipitation and the crack filling effect can be improved by co-culture. However, in this experiment, although the possibility of co-culture to increase precipitation of CaCO_3_ was investigated, incorporating bacteria spore and urea in the cementitious materials for the self-healing concrete production was not achieved. Thus, further work should therefore include to apply spore cultures as well as bacterial co-culture to the exploration of potential applications.

## 5. Conclusions

The present study investigated the effect of coculturing ureolytic and non-ureolytic bacteria on the self-filling of cement mortar. To this aim, urea–calcium lactate media containing *S. pasteurii* and *B. thuringiensis* were prepared at an inoculation ratio of 10:0, 8:2, or 5:5, to which mortar specimens with predefined cracks were exposed. The obtained results indicate enhanced CaCO_3_ precipitation by coculturing ureolytic and non-ureolytic bacteria and elucidate the principles involved in such phenomena. The findings of this study can be summarized as below:

(1) The urea–calcium lactate medium in which *B. thuringiensis* and *S. pasteurii* were co-cultured showed enhanced consumption of Ca^2+^, thereby leading to a higher extent of CaCO_3_ precipitation in comparison cultures solely containing *S. pasteurii*.
(2) The faster growth rate of the non-ureolytic organism *B. thuringiensis* compared to the ureolytic bacteria led to more rapid respiration producing CO_2_ which decrease pH and provided nucleation site that effectively enhanced CaCO_3_ precipitation.
(3) The rapid growth of *B. thuringiensis* was also effective for NB decomposition, as evidenced by the higher concentration of NH_4_^+^ in the coculturing medium, whereby production of CO_3_^2−^ and CaCO_3_ were significantly increased.
(4) The performance of the bacteria for crack filling in mortar specimens was largely dictated by the width of crack. Even though a higher amount of CaCO_3_ precipitation was achieved in the co-cultured medium, the rate of crack filling was found to be more dependent on the crack width than the co-culture ratios.

## Figures and Tables

**Table 1:** Microbial inoculation ratio by volume ratio.

Media Code	*S. pasteurii*	*B. thuringiensis*
A	10	0
B	8	2
C	5	5
